# Mortality trends in Tonga: an assessment based on a synthesis of local data

**DOI:** 10.1186/1478-7954-10-14

**Published:** 2012-08-14

**Authors:** Sione Hufanga, Karen L Carter, Chalapati Rao, Alan D Lopez, Richard Taylor

**Affiliations:** 1 Ministry of Health, Kingdom of Tonga, Tonga; 2 School of Population Health, University of Queensland, Brisbane, Australia; 3 Secretariat of the Pacific Community, Noumea, New Caledonia; 4 School of Public Health and Community Medicine, University of New South Wales, Paddington, Australia

**Keywords:** Mortality, Cause of death, Noncommunicable diseases, Medical record review, Death certification, Tonga, Pacific Islands

## Abstract

**Background:**

Accurate measures of mortality level by age group, gender, and region are critical for health planning and evaluation. These are especially required for a country like Tonga, which has limited resources and works extensively with international donors. Mortality levels in Tonga were examined through an assessment of available published information and data available from the four routine death reporting systems currently in operation.

**Methods:**

Available published data on infant mortality rate (IMR) and life expectancy (LE) in Tonga were sought through direct contact with the Government of Tonga and relevant international and regional organizations. Data sources were assessed for reliability and plausibility of estimates on the basis of method of estimation, original source of data, and data consistency. Unreliable sources were censored from further analysis and remaining data analysed for trends.

Mortality data for 2001 to 2009 were obtained from both the Health Information System (based on medical certificates of death) and the Civil Registry. Data from 2005 to 2009 were also obtained from the Reproductive Health System of the Ministry of Health (MoH) (based on community nursing reports), and for 2005–2008, data were also obtained from the Prime Minister’s office. Records were reconciled to create a single list of unique deaths and IMR and life tables calculated. Completeness of the reconciled data was examined using the Brass growth-balance method and capture-recapture analysis using two and three sources.

**Results:**

Published IMR estimates varied significantly through to the late 1990s when most estimates converge to a narrower range between 10 and 20 deaths per 1,000 live births. Findings from reconciled data were consistent with this range, and did not demonstrate any significant trend over 2001 to 2009.

Published estimates of LE from 2000 onwards varied from 65 to 75 years for males and 68 to 74 years for females, with most clustered around 70 to 71 for males and 72 to 73 for females. Reconciled empirical data for 2005 to 2009 produce an estimate of LE of 65.2 years (95% confidence interval [CI]: 64.6 - 65.8) for males and 69.6 years (95% CI: 69.0 – 70.2) for females, which are several years lower than published MoH and census estimates. Adult mortality (15 to 59 years) is estimated at 26.7% for males and 19.8% for females. Analysis of reporting completeness suggests that even reconciled data are under enumerated, and these estimates place the plausible range of LE between 60.4 to 64.2 years for males and 65.4 to 69.0 years for females, with adult mortality at 28.6% to 36.3% and 20.9% to 27.7%, respectively.

**Conclusions:**

The level of LE at a relatively low IMR and high adult mortality suggests that non-communicable diseases are having a profound limiting effect on health status in Tonga. There has been a sustained history of incomplete and erroneous mortality estimates for Tonga. The findings highlight the critical need to reconcile existing data sources and integrate reporting systems more fully to ensure all deaths in Tonga are captured and the importance of local empirical data in monitoring trends in mortality.

## Background

Mortality level is a key measure of population health, and accurate measures of mortality level by age group, gender, and region are critical for health planning and evaluation. Additionally, population health targets such as the Millennium Development Goals [1] require reliable data to measure progress. Reliable health statistics are crucial for countries like Tonga, which have limited resources and the international community of partners and donors that support health programs.

Tonga is a small Pacific Island country with 36 inhabited islands. The main island groupings are Tongatapu (including the capital Nuku’alofa) which houses 70% of the population, and Ha’apai, Vava’u, Eua, and Niua’s. Tonga has an estimated population of 103,000 (2009) [2], of which 38% are under 15 years of age [2]. Health services are provided through a national hospital, three outer island hospitals, 11 reproductive health centers, and 15 community health centers [3].

Despite the importance of mortality data, official reporting systems rarely capture every death [4]. A global assessment of mortality data in 2003 listed Tonga as having “low” quality death records, with reporting completeness estimated at 86% [4]. Substantial variation in published estimates of mortality, particularly for infant and childhood mortality, have been noted [5] and makes assessment of health trends over time difficult.

**Figure 1:**
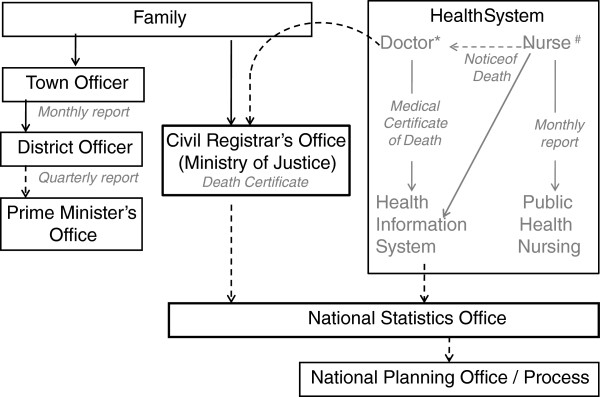
Routine mortality data reporting systems in Tonga.

Deaths in Tonga are recorded through three organizations and four systems. These are: the Health Information System (HIS) and Reproductive Health Surveillance System (RHS) operated by the Ministry of Health (MoH), the Civil Registry (CR) system managed by the Ministry of Justice, and reporting through the Prime Minister’s office (Figure 1). Coverage of these systems, in principle, is nationwide. Burials for deceased persons in Tonga occur primarily on community land that is managed by local leaders and do not generally require a formal burial permit.

Tongan legislation [6] requires families to report deaths to the Registrar or the nominated Magistrate who performs this function for outer islands. The CR office often requests that the family produce a medical certificate of death in order to complete the registration; however, this is not required by legislation. Deaths of Tongan citizens that occur overseas may be registered in the same manner.

For deaths in a hospital or health center, the attending doctor will issue a medical certificate of death. For home and community deaths, local nurses complete a “notice of cause of death,” which is used by the certifying doctor to complete a medical certificate of death [7]. A copy of this certificate is sent by the hospital to the Health Information Section, where the data are coded and entered onto the HIS database. District nurses are also required to submit monthly reports, including details of known deaths. These forms are forwarded to the reproductive health office. Data are aggregated by hand for inclusion in the annual report.

Reporting to the Prime Minister’s office is primarily for the purposes of maintaining the electoral roll, although deaths at all ages are collected. Reports are submitted monthly by locally elected community leaders [8].

In this study, mortality levels in Tonga were examined through an assessment of available published information and data available from the four routine death reporting systems in operation. Empirical data from these systems were reconciled to derive summary measures of mortality, including infant mortality rate (IMR), childhood mortality (probability of dying before the age of 5, _5_q_0_), average life expectancy at birth (LE), and adult mortality (probability of dying between ages 15 and 59, _45_q_15_). The reconciled dataset was further examined for potential under-enumeration of deaths using both a Brass analysis [9] and capture-recapture techniques [10], and the plausibility of the derived mortality estimates is discussed.

## Methods

Measures included in the analysis and reported in this paper are from two sources: (1) previously published reports [11-60] and (2) empirical data on deaths by age group, sex, and period for 2001 to 2009 obtained from local sources. As key informant interviews with the registrars’ office indicated, most overseas deaths registered are for Tongan residents. All deaths registered in Tonga were included.

### Published data collection and analysis

#### Data collection

Available published secondary data [11-60] on mortality in Tonga were sought through direct contact with the government of Tonga and international and regional organizations with an interest in health or mortality in Tonga. A literature search was conducted, including an internet search of websites for United Nations agencies (UNDP, UNICEF, UNFPA, and WHO); regional institutions such as the Secretariat of the Pacific Community (SPC), the World Bank (WB), and the Asian Development Bank (ADB); and non-governmental organizations.

#### Data assessment

**Figure 2:**
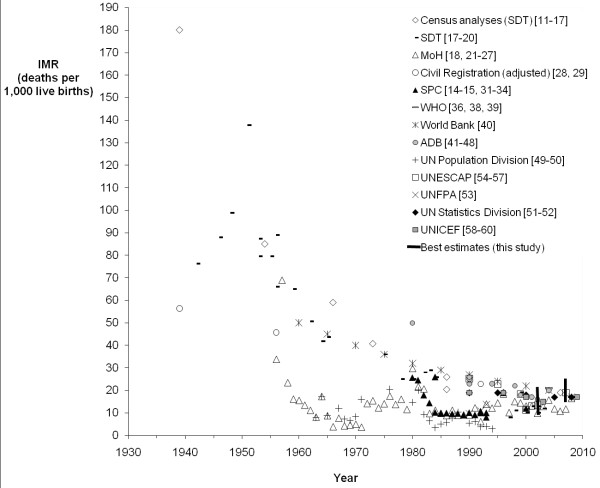
**Published estimates of Infant Mortality Rate (IMR) by data source, 1939–2009.** SDT = Statistics Department of Tonga, MoH = Ministry of Health, SPC = Secretariat of the Pacific Community, WHO = World Health Organization, ADB = Asian Development Bank. The range of best estimates from this study (Table 2) for 2001–2004 and 2005–2009 is shown at 2002 and 2007, respectively.

**Figure 3:**
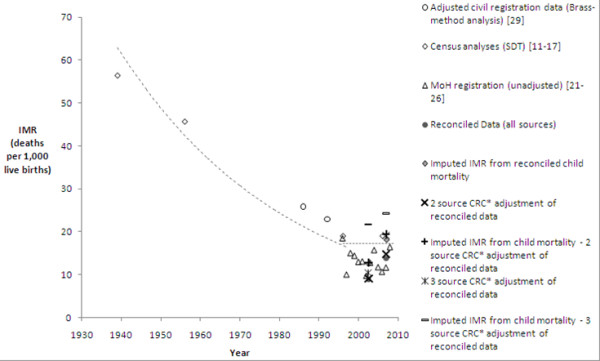
**Credible published and empirical estimates of IMR by source and method of adjustment, 1939–2009.** SDT = Statistics Department of Tonga, MoH = Ministry of Health, CRC = Capture-recapture analysis y = 200 + 21e^-0.023x^ / y = 0.0025x + 12.16 (fitted to credible estimates excluding MoH estimates as described in the methods).

IMR and LE were chosen as measures of all-cause mortality due to availability. IMRs from published reports were graphed over time by major source of data (Figures 2 and 3). Data sources were assessed for reliability and plausibility of estimates on the basis of method of estimation, original source of data, and data consistency. Unreliable sources were censored from further analysis if they met any of the following criteria: (a) data were derived, or were considered likely to have been derived, from models assuming a given improvement by year, as evidenced by a perfectly linear improvement in LE or IMR by year; (b) multiple incompatible estimates were given by the source for a single year or adjacent years; (c) source data included implausible estimates (based on equivalent measures for developed countries); (d) calculations were based on uncorrected vital registration data known to be significantly underreported or with no assessment of reporting completeness.

**Figure 4:**
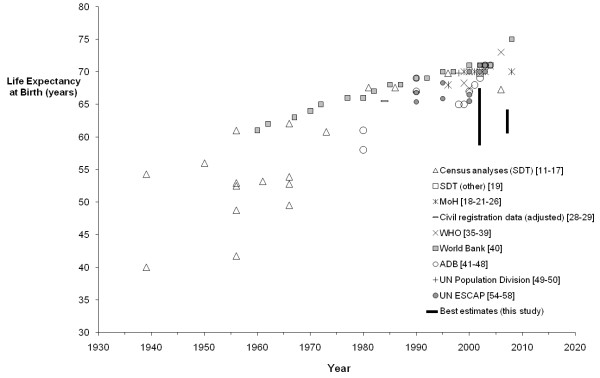
**Published estimates of life expectancy at birth by source, males, 1930–2009.** The range of best estimates from this study (Table 2) for 2001–2004 and 2005–2009 is shown at 2002 and 2007, respectively.

**Figure 5:**
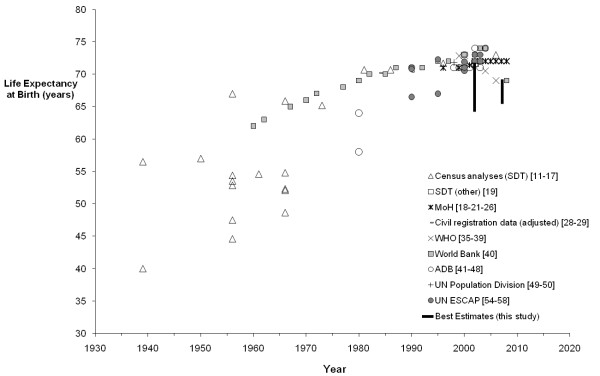
**Published estimates of life expectancy at birth by source, females, 1930–2009.** The range of best estimates from this study (Table 2) for 2001–2004 and 2005–2009 is shown at 2002 and 2007, respectively.

An exponential trend was fitted to the average IMRs of each year from data remaining after censoring (Microsoft Excel). A linear trend was fitted to IMRs post 1996, excluding the MoH estimates, which were retained after censoring but later shown to be incomplete based on the empirical data analysis. LE from all sources were graphed over time by major source of data (Figures 4 and 5), with unreliable estimates (based on the same criteria as described for IMR) censored from further analysis. Insufficient data remained after censoring to fit a trend curve.

### Empirical data collection and analysis

#### Data collection

Data for 2001 to 2009 were obtained from both the HIS (derived from medical certificates of death) and CR. Data from 2005 to 2009 were obtained from the RHS records (community nursing reports), and data for 2005 to 2008 were obtained from the Prime Minister’s office (Table 1). Additional years from the RHS and Prime Minister’s office were not available at the time of data collection. Key variables collected were: island of residence, data source, full name, sex, date of birth, date and place of death, and age at death. Population by age group, sex, and island was derived from the 1996 [14,15] and 2006 [16,17] censuses using exponential interpolation to provide estimates for intermediate and additional years. Total births for 2005 to 2009 were obtained from the MoH annual reports for calculation of IMR [21].

**Table 1:** Reported deaths by source, all Tonga: 2001-2009

**Year**	**Civil Registration**	**HIS (death certificates)**	**RHS (community nursing reports)**	**Prime Minister’s office (town and district officer reports)**	**Reconciled data (all sources)**
2001	473	567			605
2002	446	588			627
2003	434	598			629
2004	418	543			574
2005	406	534	133	36	657
2006	470	493	146	44	713
2007	400	526	144	74	735
2008	398	484	543	60	957
2009	468	567	393		836

HIS = Health Information System, RHS = Reproductive Health System.

#### Reconciliation of reported deaths

Each death was assigned a unique number. Records were reconciled to create a single list of unique deaths indicating source(s) in which each was recorded. Investigators manually matched deaths through a repeated sorting of lists by key criteria, and the final list of unique deaths was reviewed by two investigators (SH, KC). Criteria were established for records to be considered a match: (1) same surname (minor variations in spelling, variations in name order, or a phonetic match were allowed), (2) same first name (again, minor variations allowed), (3) age at death within one year, (4) date of death within the same month, and (5) either same place of residence or same place of death. Records were considered to match if they met three criteria including surname or four criteria excluding surname.

Deaths with missing ages were then redistributed according to imputed age distributions for that source. Deaths only reported through the Prime Minister’s Office (Table 1) were excluded from the reconciled data, as the limited information provided and poor data quality meant that it was not possible to determine whether these were additional deaths, or whether there was simply insufficient information to identify an existing match.

The following measures of mortality were calculated using standard methods [61]: age-specific mortality rate, IMR (probability of dying before 1 year of age), childhood mortality (probability of dying before 5 years of age), adult mortality (probability of dying between 15 and 59 years of age inclusive), and LE. Childhood (<5 years) mortality was also calculated based on a regression of the IMR as a proportion of childhood mortality against childhood mortality from international estimates for 2006 [62]. Estimated reporting completeness by data source was calculated using the total reconciled deaths.

#### Assessment of completeness of reconciled data

Reconciled data were assessed for under enumeration of deaths by (1) the Brass growth-balance method, which compares the age distribution of reported deaths with the expected age distribution of deaths based on the observed population age-sex structure (assuming a closed population), applied to ages 25 to 64 years [61,63] and (2) capture-recapture techniques, which use the proportion of reported deaths recorded by each combination of sources to estimate the number of unreported deaths [10,63]. Data from the Prime Minister’s office were excluded from the capture-recapture analysis as very few deaths were reported (Table 1), and these were from a very small area of Tonga, thus violating the assumption of equal catch ability for each death [10].

A two-source capture-recapture analysis was conducted for HIS data against CR data for 2001 to 2004. A key assumption in a two-source analysis is that sources are independent. Data from the HIS and RHS for 2005 to 2009 were therefore reconciled as a single source (henceforth referred to as MoH data) as both systems are operated by the MoH and therefore demonstrate strong interdependency. MoH data was then assessed against CR data for 2005 to 2009. Confidence intervals (CIs) were calculated using the maximum likelihood estimator [10].

Three-source capture-recapture analysis allows for pair-wise dependence between sources [10,64] and used HIS, RHS, and CR data separately for 2005 to 2009. Each three-source analysis results in eight possible models [10,64,65]. Models that did not account for dependence between the two MoH sources were excluded based on the evaluation of the death recording systems with final model selection based on statistical criteria such as the significance of associations between sources (p-value) and goodness-of-fit criteria (Bayesian Information Criterion and the Akaike Information Criterion) [10,65]. HIS data for 2001 to 2004 were adjusted by reporting completeness for the HIS data for 2005 to 2009 to generate an adjusted estimate for this period based on the three-source analysis. Unreported deaths were estimated for total deaths, with subgroup analyses for sex, age, and island group for both the two- and three-source capture-recapture analyses to identify variation in reporting patterns.

Reporting completeness for Tongatapu was applied to all islands to derive adjusted estimates of total deaths, as most deaths are from Tongatapu and it was anticipated that data from the main island group (where data are checked more regularly) should be of the highest quality and would therefore be less likely to be under matched due to data recording issues. Adjusted measures of LE and IMR were then compared with reconciled data and credible estimates from the published data.

## Results

### Analysis of published data

Thirteen sources of published mortality estimates were found for Tonga, with 10 of these sources reporting LE. There was very little information on methods of data collection and analysis available in the published sources.

#### Infant mortality rate

Published IMR estimates varied significantly through the late 1990’s when most estimates converge to between 10 and 20 deaths per 1,000 live births. Despite the variation, it is apparent IMR has fallen substantially over the period investigated and is now relatively low (Figures 2 and 3). Four sources remained after applying the censoring criteria, including MoH estimates from reported deaths. Significant variation remains in recent estimates despite censoring. Based on credible published estimates, IMR for Tonga appears relatively stable for recent years: between 10 and 19 deaths per 1,000 live births.

#### Life expectancy

Ten sources of published estimates of LE were found for Tonga since 1939. There was a wide variation found in published estimates of LE (around 20 years’ difference for a single year for each sex), as shown in Figures 4 and 5. Eight sources were censored from further analysis, leaving only uncorrected MoH data and an earlier study that used CR data for 1982 to 1986 and 1987 to 1992 adjusted for completeness [19]. After censoring, published estimates of LE were 69.6 years for males and 72.9 years for females for 2005 to 2008 (Table 2).

**Table 2:** Summary estimates of mortality (all Tonga) by method and period

**Mortality dataset**		**Period**	**IMR /1,000**	**IMR /1,000 imputed from child mortality**	**Child mortality <5 years /1,000**		**Adult mortality (%)**		**Life expectancy at birth (years)**	
					**M**	**F**	**M**	**F**	**M**	**F**
Credible published sources	Civil Registry data (adjusted by Brass analysis) [29]	1982-1986	25.9	-	-	-	-	-	66.3	69.5
		1987-1992	22.9	-	-	-	-	-	68.1	72
	Ministry of Health data only (unadjusted) [5]	2005-2008	16.4	-	-	-	18.4	19.1	69.6	72.9
Reconciled data		2001-2004	8.9	12.6	16.7	11.5	21.8	15.5	67.7	71.7
		2005-2009	13.7	18.2	24.5	19.5	26.7	19.8	65.2	69.6
Adjusted reconciled data	Adjusted by Brass analysis (>5 years)	2001-2004	NA	NA	NA	NA	21.6	22.1	67.9	68.3
		2005-2009	NA	NA	NA	NA	26.7	23.6	65.2	67.8
	Adjusted by two-source CRC analysis (all ages)	2001-2004	9.1	12.8	17.0	11.8	22.2	15.8	67.5	71.6
		2005-2009	14.7	19.4	26.6	20.7	28.6	20.9	64.2	69.0
	Adjusted separately for three-source CRC analysis for 0–4 years and remaining deaths by gender	2001-2004†	10.4	21.7	32.1	21.8	39.4	29.8	58.8	64.2
		2005-2009	19.1	24.7	34.8	27.6	36.3	27.7	60.4	65.4
Final mortality estimates		2001-2004	9.1 – 21.7		17.0 – 32.1	11.8 – 21.8	22.2 -39.4	15.8 – 29.8	58.8 - 67.5	64.2 - 71.6
		2005-2009	14.7 – 24.7		26.6 – 34.8	20.7 – 27.6	28.6 – 36.3	20.9 – 27.7	60.4 - 64.2	65.4 – 69.0

CRC = Capture-recapture analysis.

Credible estimates of IMR are shown in Figure 3.

†Estimates generated by applying reporting completeness for HIS data from 2005 to 2009 three-source analysis (final disaggregation by 0 to 4 years, and remaining deaths by gender) to HIS data for 2001 to 2004.

### Analyses of reported deaths

#### Reconciled data

There were 3,874 unique deaths recorded for Tonga between 2005 and 2009 (Table 1) and 2,435 deaths recorded between 2001 and 2004. The MoH recorded 3,361 (87%) of total reconciled deaths for 2005 to 2009, while the CR recorded 2,128 deaths (55%). The Prime Minister’s office recorded 214 deaths for 2005 to 2008, of which only 14 were not able to be matched with another source.

From the reconciled deaths, IMR was directly calculated for 2005 to 2009 as 14.7 deaths per 1,000 live births, with childhood mortality as 24.5 deaths per 1,000 live births for males and 19.5 for females. Imputation of IMR from these levels of childhood mortality yielded an estimate of 18.2 deaths per 1,000 live births. Adult mortality is estimated at 26.7% for males and 19.8% for females (Table 2). LE at birth (2005 to 2009) was 65.2 (95% CI: 64.6 – 65.8) years for males and 69.6 (95% CI: 69.0 - 70.2) years for females. LE is lower than published estimates presented above and substantially lower than published estimates that were censored. If overseas deaths are excluded to minimize the possibility of nonresidents being included in the analysis, there is less than 0.5 years increase in LE for both males and females. There is no change to IMR.

Reporting completeness for individual data sources (2005 to 2008), as determined from a comparison with total reconciled deaths, is estimated at 55% for CR, 67% for HIS, 35% for RHS, and 87% for the combined MoH data. For Tongatapu, reporting completeness was estimated at 56% for CR, 70% for HIS, 42% for RHS, and 91% for the combined MoH data (Table 3).

**Table 3:** Estimated reporting completeness (%) of deaths (all ages, both sexes) by source and method of assessment for Tongatapu

**Method of assessment**	**Years**	**Completeness (%) and 95% confidence intervals**				
		**Reconciled death data**	**Civil registration of deaths**	**HIS (death certificates)**	**RHS (community nursing reports)**	**MoH (combined)**
Comparison against reconciled death data	2001-2004	-	70	96	NA	NA
	2005-2009	-	56	70	42	91
Brass growth-balance assessment	2001-2004	96 (M) / 63 (F)	-	-	-	-
	2005-2009	>99 (M) / 82 (F)	-	-	-	-
Two-source capture-recapture analysis	2001-2004	98 (97 – 99)	69 (68–70)	94 (93 – 95)	NA	NA
	2005-2009	93 (91–95)	53 (52–54)	66 (65–67)	40 (39–40)	84 (83–86)
Three-source capture-recapture analysis (aggregated ages)	2005-2009	68 (63–73)	37 (34 – 40)	46 (43 – 50)	28 (26 – 30)	63 (58 – 67)
Three-source capture-recapture analysis (final disaggregation*)	2005-2009	69 (57 – 77)	38 (31 – 42)	47 (39 – 52)	28 (23 – 31)	63 (52–70)

95% confidence intervals shown in parentheses where applicable.

HIS = Health Information System, RHS = Reproductive Health System, MoH = Ministry of Health (HIS and RHS data combined).

* Capture-recapture analysis disaggregated by the following subgroups: 0 to 4 years (both sexes), males 5 years or older, and females 5 years or older.

#### Brass analysis

Brass analysis of the reconciled data estimates that reporting completeness for 2005 to 2009 (all sources) is >99% for males and 63% for females; however, the disparity between these estimates is not plausible. For Tongatapu only, reporting completeness was estimated as >99% for males and 82% for females. The Brass analysis plot of partial births against partial deaths for males (for all deaths and Tongatapu only) did not readily allow a straight line to be fitted regardless of age groups selected, indicating this estimate of completeness is unreliable.

#### Capture-recapture analysis

The overall two-source analysis of aggregated health records and civil registration records estimated a total of 2,491 (95% CI: 2465–2517) deaths in Tonga for 2001 to 2004 and 4,400 (95% CI: 4295–4506) deaths for 2005 to 2009. Compared to these estimates, reconciled data from both the CR and health reporting systems were found to be 98% (95% CI: 97–99) complete for 2001 to 2004 and 88% (95% CI: 85–90) for 2005 to 2008 for both sexes. For Tongatapu only, the reconciled data were estimated as 98% (95%: CI 97–99) complete for both sexes for 2001 to 2004 and 93% (95%: CI 89–95) complete for 2005 to 2009 (Table 3). Recording completeness varied by age, with child deaths less likely to be recorded than adult deaths through CR. However, aggregation of subgroup analyses within strata of age and island group provided summary measures of mortality, which differed little from the overall aggregated estimates. Reporting completeness for individual data sources (2005 to 2008) in Tongatapu, as determined by the two-source analysis, is estimated at 53% for CR data, 66% for HIS data, 40% for RHS data, and 84% for the combined health data (HIS plus RHS).

A three-source analysis was performed for HIS, RHS, and CR data for 2005 to 2009. Estimates of total deaths ranged from 4,404 to 6,695 based on aggregate (total) data, depending on the model selected. The model selected as the best fit was that which included dependence between the HIS and RHS and between the HIS and CR. This is consistent with the assessment of the death reporting system. Based on this model, the reconciled data are estimated to be 58% (95% CI: 52 – 63) complete. If only Tongatapu is included, the reconciled data are estimated to be 70% (95% CI: 63–76) complete for males and 67% (95% CI: 58–74) complete for females. As reporting patterns varied by age (particularly for very young deaths) and sex, final estimates of completeness from the three-source analysis were derived from models of best fit for disaggregated data for children aged 0 to 4 years (both sexes) and the remaining ages by sex. Based on these models, the reconciled data for Tongatapu were found to be 70% (95% CI: 32–91) complete for children aged 0 to 4 years, 69% (95% CI: 61–76) complete for males 5 years and over, and 68% (95% CI: 59–75) complete for females 5 years and over. Adjusted estimates are shown in Table 2.

#### Final estimates of mortality

Both the Brass method analysis and capture-recapture analyses indicate that deaths remain under enumerated following data reconciliation. The Brass method, however, provided implausible results, due most likely to the sensitivity of this method to high levels of migration and small numbers [66]. The two-source capture-recapture analysis is less sensitive but is known to be affected by probable positive dependence between the civil registry and health data due to registration practices; therefore, it is expected to overestimate reporting completeness. As such, the two-source analysis provides a plausible lower limit to mortality levels and subsequent upper limit for LE. Equally, the three-source analysis allows for this dependence and can therefore be used to set a plausible upper limit for mortality levels and hence a lower limit for LE. Final estimates for 2005 to 2009 place IMR between 14.7 to 24.7 deaths per 1,000 births, with the best estimate for this period at 19 deaths per 1,000 births, as estimated from childhood mortality from the two-source capture-recapture analysis, direct calculation from the three-source analysis, and the 2006 census. Final estimates place the plausible range of LE at 60.4 to 64.2 years for males and 65.4 to 69.0 years for females. Adult mortality is estimated to be between 28.6% to 36.3% for males and 20.9% to 27.7% for females.

## Discussion

These findings demonstrate that there has been a sustained history of erroneous mortality estimates for Tonga, with many published estimates of LE and IMR implausible and presented without an indication of the method used. Many of the IMR estimates presented prior to 2000 (Figure 3) fall below current levels of IMR in Australia (IMR of 5.0 in 2007 [58]) and New Zealand (IMR of 6.0 in 2007 [58]). Credible data sources demonstrate that IMR has declined steadily to below 20 deaths per 1,000 live births, fluctuating in recent years between 10 and 19 deaths per 1,000 live births. Although higher than previous MoH estimates of IMR, reconciled estimates of IMR are consistent with this range, while several adjusted estimates are slightly higher. The range of plausible estimates suggests that progress toward the Millennium Development Goal targets has been minimal, with best estimates from the empirical data from 2005 to 2009 at very similar levels to census estimates for 2006. Although IMR demonstrates no significant trend since the late 1990s, at these levels it is a minor influence on the overall LE [61].

Published estimates of LE from 2000 onwards varied from 65 to 75 years for males and 68 to 74 years for females, with most clustered around 70 to 71 for males and 72 to 73 for females. Very few of the published estimates were assessed as reliable, with only MoH data remaining for recent years. These MoH data estimated LE for 2005 to 2008 as 69.6 years for males and 72.9 years for females. The 2006 census estimates of 67.3 years for males and 73.0 years for females were censored from the final results as LE was calculated from registration data adjusted using a model with a single input parameter (childhood mortality) [16,17].

The reconciled empirical data for 2005 to 2009 produces an estimate of LE of 65.2 years (95% CI: 64.6 - 65.8) for males and 69.6 years (95% CI: 69.0 – 70.2) for females, which is several years lower than both the MoH and census estimates. These findings demonstrate that LE is substantially lower than previously reported for Tonga. Further, the reconciled data indicate LE may have fallen from 2001 to 2004, although this is not evident in the range of plausible published estimates for each period.

Although there remains some uncertainty regarding the extent of underreporting to all data sources, the data collection systems and results of the assessments presented here indicate that even reconciled data under enumerated. The Brass analysis, particularly for males, appears significantly affected by migration, which is quite high in Tonga (−16.58 per 1,000 population in 2010 [67]). However, the capture-recapture assessments presented here allow limits of plausibility to be established. The two-source analysis for 2005 to 2009 accounts for the major dependency between the HIS and RHS by combining these data; however, there is also probable positive dependency between the HIS and CR due to the Civil Registry requesting medical certificates of death. As such, this analysis would overestimate reporting completeness and underestimate the total deaths, thus providing a plausible upper limit for LE. The three-source capture-recapture analysis allows these dependencies to be taken into account, and can therefore be used as a lower limit for LE, producing similar results to the Brass analysis for females. As migration data for Tonga are not complete, it is not possible to use changes in population recorded through the census as an alternative method for assessing reporting completeness for deaths.

The low LE is clearly being predominantly driven by adult mortality. Even prior to adjustment for undercounting, adult mortality based on reconciled data in Tonga is roughly three times that seen in Australia, where there is a probability of dying of 8.9% for males and 5.1% for females in 2002 to 2004 [68], or New Zealand, where there is a probability of dying of 9.7% for males and 6.4% for females in 2002 to 2004 [69]. The high adult mortality is consistent with the limited information available on noncommunicable disease risk factor prevalence available for Tonga. Provisional results from a 2004 survey found that 69% of adults were obese (body mass index ≥ 30 kg/m^2^), 23% of adults were affected by hypertension (systolic blood pressure ≥ 140 mmHg and/or diastolic blood pressure ≥ 90 mmHg or currently on medication), and 18% reported to be diabetic (self-report) [70]. In 2004, Tonga acted to recognize the extensive community burden on noncommunicable diseases by developing the first non-communicable disease strategy in the Pacific region [71]. This pattern of high adult mortality limiting LE is also consistent with recent findings elsewhere in the Pacific, such as in Fiji and Nauru [72,73].

Difficulties encountered in assessment of published data were the lack of information concerning primary data sources, methods of calculation, and assumptions. In order to deal with this, exclusion criteria were specified and unreliable estimates censored from further analyses. The matching criteria used to reconcile the sources of death reporting and multiple checks of the matching process minimize the risk of significant errors in the reconciliation of the data sources. These criteria were developed and tested to reduce the risk of matching records being missed, with the matching process checked by two investigators (SH, KC). Removing the Prime Minister’s office data from the analysis further reduced the potential for under matching due to incomplete data, which would have lead to overestimation the number of deaths not reported [10,63]. Completeness estimates for the main island group of Tongatapu, as the best quality subset of data, were also applied to all data to minimize the potential for overestimating unreported deaths due to misalignment in the populations covered by each source or poorer data quality from the outer islands.

The wide range of plausible estimates for the period 2001 to 2004 limit any assessment of trends between the two time periods investigated. This uncertainty is largely a result of less data being available for these years. However, as MoH efforts to improve reporting began after this period, HIS data for 2001 to 2004 could reasonably be expected to be less complete than in 2005 to 2009. This is reflected in the increase in the total reported deaths per year in the reconciled data between the two periods, as shown in the results. The true level of mortality is therefore likely to fall closer to the high end of the range of plausible mortality estimates for 2001 to 2004 (and therefore the lower estimates of LE) as derived by applying reporting completeness for the HIS from the three-source assessment for 2005 to 2009. While there have been very few studies on reporting completeness in Tonga, our findings of reporting completeness for the reconciled data are consistent with a 2003 evaluation of mortality data in Tonga based on data obtained by the World Health Organization. This report found Tonga to have “low” quality mortality and cause of death data, with completeness estimated at 86% and coverage estimated at 70% [4], compared with an estimated completeness for the reconciled data between 68% and 93% for 2005 to 2008, as found in this study.

## Conclusions

LE for Tonga for 2005 to 2008 is estimated to be between 60.4 to 64.2 years for males and 65.4 to 69.0 for females, well below previously published estimates. The low LE, at a relatively low IMR and high premature adult mortality, suggests that non-communicable diseases are having a profound limiting effect on health status in Tonga.

These findings demonstrate that much of the mortality data that have been previously available to health policy makers in Tonga as well as to international donors and agencies has been misleading, potentially masking the urgency of the public health action need to address adult premature mortality in Tonga. Additionally, the findings highlight the critical need both to reconcile existing data sources and integrate reporting systems more fully to ensure all deaths in Tonga are captured, as well as the importance of analysis of local empirical data in monitoring trends in health status. While census estimates for IMR were found to be consistent with best estimates from the routinely reported data, the census was found to have underestimated adult mortality and therefore overestimated LE.

## Competing interests

AL is one of the Editors-in-Chief of *Population Health Metrics*.

## Authors’ contributions

SH conducted the system review, supervised the data collection and collation processes, designed the matching criteria, conducted the data matching, managed the ethics process and coordination with data collection agencies, performed the statistical analysis for the two-source analysis of the HIS and PM data for 2005 to 2009, completed the search for published estimates, assessed published data sources for reliability, revised the manuscript critically, and made substantial contributions to the interpretation of data. KC oversaw the project, conducted the system review, oversaw the design and testing of matching criteria, checked the matching process, assisted with data matching for the civil registration data against other sources, performed the statistical analysis for both the two-source analysis for 2001 to 2004 and the three-source analysis, including the civil registry data and final model selection including interpretation of final results and selection of final estimates, supervised the other analysis, and drafted and led the revision of the manuscript. CR revised the manuscript critically for important intellectual content. ADL and RT conceived and coordinated the broader study under which this project was undertaken, reviewed and contributed significantly to the interpretation of results, and revised the manuscript critically for important intellectual content. RT also generated the regression models to estimate IMR from childhood mortality. All authors read and approved the final manuscript.
